# Mental health of returnees: refugees in Germany prior to their state-sponsored repatriation

**DOI:** 10.1186/1472-698X-8-8

**Published:** 2008-06-12

**Authors:** Ulrike von Lersner, Ulrike Wiens, Thomas Elbert, Frank Neuner

**Affiliations:** 1 Psychotrauma Research- and Outpatient Clinic for Refugees, University of Konstanz, Germany

## Abstract

**Background:**

Many refugees live for years in exile. The combination of stress in the host country, together with long-term effects resulting from traumatic stress usually experienced in the home country may affect mental health. Little is known, to what extent these and other factors promote or stall the willingness to return to the country of origin. Here, we investigate, as an example, refugees who will return to their country of origin after having lived in exile in Germany for some 11 years.

**Objective:**

What is the mental health status of returnees before the actual return who have been living in exile for an extended period? We also asked, what are the current living conditions in Germany and what are the motives for and reasons against a voluntary return to the country of origin?

**Methods:**

Forty-seven participants of programs for assisted voluntarreturn were interviewed about their present living situation, their view regarding their home country and voluntary return. These findings were compared to a group of 53 refugees who had decided to remain in Germany (stayers). Participants were recruited by means of advertisements posted in refugee centres, language schools, at doctors' offices and in organisations involved in the management of voluntary return in Germany. The prevalence of psychiatric disorders among respondents was tested using the structured interview M.I.N.I. The Posttraumatic Stress Diagnostic Scale (PDS) was used to assess PTSD in more detail and EUROHIS was applied to measure the subjective quality of life of participants.

**Results:**

We found a prevalence rate of 44% psychiatric disorders in the group of returnees and a rate of 78% in the group of stayers. We also recorded substantial correlations between the living situation in Germany, disposition to return and mental health. In almost two thirds of the participants the decision to return was not voluntary but strongly influenced by immigration authorities. The most important reason for participants to opt for a stay in Germany were their children, who have been born and raised in Germany.

**Conclusion:**

Psychological strains among the study participants were very high. Traumatic stress, experienced during war and refuge, has left the victims vulnerable and not well equipped to cope with post-migration stressors in exile. It is noteworthy that the majority returned under pressure of the immigration authorities. The fear of an uncertain future after the return was substantial. These factors should be taken into account in programs designed to assist returnees, including those that offer support after return to the country of origin.

## Background

Violent conflicts around the world are sending large numbers of refugees and asylum seekers into flight. Moreover, economic disadvantage – predominantly in resource-poor regions – is causing many to migrate to industrialised countries. As a consequence, welfare and economic systems of the receiving countries are reaching their limits of capacity (or willingness) to integrate incoming migrants. This is true for Western countries but even more so for poor countries like, e.g., Uganda which not only accept a higher proportion of refugees but also are forced to cope with huge numbers of internally displace people. In order to reduce the challenge on the capacity for integration as well as for the social security, welfare and health systems, the concept of 'voluntary return' with the goal of a 'humanitarian reintegration' into the country of origin has developed into one of the central instruments of both European and German migration policy [9]. UNHCR's Handbook on Voluntary Repatriation defines voluntariness as "absence of any physical, psychological or material pressure [53]. One of the most important elements in the verification of voluntariness is the legal status of the refugee in the country of asylum."

In the definition by the International Organisation for Migration (IOM) it is stated: "...that voluntariness exists when the migrants' free will is expressed at least through the absence of refusal to return, e.g. by not resisting boarding transportation or not otherwise manifesting disagreement" [14].

Entenmann and ZIRFcounselling give an overview on Voluntary Assisted Return Programs (VARP) in Germany and the European Union [6,17]. Despite a large variety in the specific implementation there are two core requirements included in all programs of assisted voluntary return: 1) the voluntariness of the individual to return to the country of origin and 2) assistance in the homeland guaranteed by the organisation (summarised in a paper of the Bundesarbeitsgemeinschaft der Freien Wohlfahrtspflege) [5].

### Motivation to return

The concept of 'voluntary return' as one possible way of handling the solution to the refugee question is not new. However, little research has been carried out from an empirical perspective. Of the existing empirical studies, the majority concentrate on the motives of refugees and migrants to return to their country of origin [21,39,49,51]. Regardless of the sample group, these studies have all reached similar conclusions. Many allocate the motives for return into three categories: 1) familial-personal reasons, 2) economic-occupational reasons and 3) social-patriotic reasons. Another way these studies classify motives for return is through so-called 'Push- Pull Factors'. 'Pull Factors' attract the potential returnee away from the receiving country and back towards their country of origin. Some of these 'Pull Factors' include family ties, homesickness and a sense of national loyalty.

Coupled with these are 'Push Factors' which make a prolonged stay in the receiving country unattractive and pressure – or push – the potential returnee out. Such factors include insufficient monetary funds, insecure visa or residential status, discrimination, language barriers and inability to adjust to the living conditions in the receiving country [12,39]. The 'Push-Pull Factors' can, in turn, be assigned to the three categories outlined above [30].

The studies in question repeatedly demonstrate that the 'Pull Factors' play a larger role in the decision to return voluntarily. Moreover, they show that there is generally no single key motive for the decision to return. Instead, the decision is influenced by a number of different factors, which may vary in importance for the potential returnee over time.

Thus, the study presented here, used an entire set of 'Push-Pull-Factors' which were designed with the aforementioned categories in mind.

### Mental health of refugees

Post-traumatic Stress Disorder (PTSD), which can be caused by experiences of violence and war, is a disorder frequently diagnosed in refugee populations and has been of growing interest in empirical research in recent years [13,26,31,37]. In a meta-analysis in refugee populations, which included 7000 refugees living in Western countries prevalence rates of 9% for PTSD and 5% for depression were found [18]. The comorbidity of these diagnoses was very high in this sample.

The reported rates of PTSD in refugees from the former Yugoslavia, who are now living in exile, lie between 30 and 60% [2,50,55,56]. The percentage of trauma-related mental disorders and functional impairment reported naturally varies according to the number of traumatic stressors influencing the population as well as the political circumstances they are in [20,32,34,38]. This explains the large variances between the different studies regarding the prevalence of PTSD.

As shown in a study by Hunt and Gakenyi, emotional distress as well as mental disorders are more frequently observed in refugees who had left their own country compared with people who remained in their home region or were internally displaced [25]. As Marshall et al. have shown, this difference persists even after 20 years in exile [33].

Unfortunately, the concentration of attention on PTSD sufferers has been to the detriment of refugees suffering from other psychological disorders like depression, as their illnesses are often left out of scientific – and in turn political – debate. On the other hand, an increasing number of researchers understand that aside from traumatic experiences there are other factors, so called post-migration factors, which cause further psychological distress in exiled people. Post-migration factors play an important role in both development and the perpetuation of mental disorders such as anxiety disorders, somatization disorders and especially depression [27,28,55]. Another study examined the influence of the living conditions of refugees in relation to the development and perpetuation of mental disorders [47]. They found that post-migration factors (such as difficulties in integrating, a loss of contact with one's cultural roots), amount to 14% of the variance of the PTSD-pathology in comparison to 20% variance of pre-migration factors. A significant correlation between stressors in exile (such as low activity levels or social isolation) and symptoms of depression, was also found in Bosnian refugees [34]. In a longitudinal study, Ruf et al. showed that the attainment of permanent residential status led to a decrease in symptoms of depression but had no influence on PTSD [41].

### Mental health of returnees

In recent years research on the mental health of returnees has gained increasing attention. Roth et al. interviewed refugees from Kosovo in Sweden shortly after their arrival, following-up with interviews three, six and eight-teen months thereafter [40]. At the time of arrival 37% of the refugees were diagnosed with PTSD. Eight-teen months later, part of the group had returned to Kosovo. 52.4% of those who had returned were diagnosed with PTSD. The rate in the group that stayed in Sweden was 85.3%. In a similar study, Toscani et al. examined the living conditions of returnees from Switzerland to Kosovo and found a PTSD rate of 25% in this group. 65% of the Kosovo returnees were living in extreme poverty and suffered generally from poor health [52]. This study demonstrated a negative correlation between the length of the returnees' time in exile and their mental health after their return home. The authors of both studies suggest the reason for these findings is the additional post-migration distress experienced in exile [44,45]. In contrast, Sundquist et al. also carried out a longitudinal study on Chilean and Uruguayan refugees who had been in exile in Sweden [48]. Those who returned home suffered more in terms of mental health and integration than those who remained in Sweden. Level of mental stress, discrimination and insecurity in everyday life were also higher among returnees. The contradictory results presented here reflect the lack of definite empirical information regarding the mental health of refugees in the return process.

### Project context

The main objective of Voluntary Assisted Return Programs (VARP) is to reduce the number of migrants living with unstable visa status in receiving countries. On the other hand, these programs also aim for the sustainable reintegration of returnees into their home country.

Until now, little empirical data has been collected on the sustainability of such programs. Also there is no information on the impact of the return process on the mental health of the persons concerned. This information is crucial if one is to help guide the successful reintegration of returnees into the society of their home country.

The study presented here describes the second part of a longitudinal survey. The overall goal of the survey is to analyse the phenomenon of 'voluntary return', its potential and its limitations from a psychological perspective. Thereby we focussed on people, who arrived to Germany as refugees and/or asylum-seekers. The first part of the survey investigated the motives of refugees from the former Yugoslavia who did not want to return home but rather preferred to remain in the receiving country which in this case was Germany [55]. For our purposes here, this group will be referred to as the 'stayers'.

In the second part of the survey – described in this paper – we interviewed participants of VARP before their return and compared their answers to the data collected from the stayers. We investigated the present living conditions, mental health, quality of life as well as the motives for or against voluntary return in both groups. The data collected during this investigation will help improve our understanding of the situation in which returnees find themselves. This in turn may result in the creation of more reliable, applicable and/or effective services to meet their needs as well as those of the host countries.

The final phase of our study, will examine the living conditions of returnees nine months after return. Those results will be published in the near future.

## Methods

### Experimental design

Participants were recruited by means of advertisements posted in refugee centres, language schools and at doctors' offices. Further every single organisation involved in the management of voluntary return or VARP in Germany was contacted. Participants were recruited and interviewed between June 2005 and March 2007. In total, for the group of returnees 45 organisations were contacted, of which ten referred clients to us. Seven organisations refused to cooperate, citing political reasons. Three organisations referred clients too close to their scheduled return, making it impossible for them to be interviewed. The remaining organisations did not have any clients who fit our inclusion criteria, i.e. who returned to the former Yugoslavia, Turkey or Iraq.

For the comparison group (stayers) the sub-set of 21 institutions was contacted, of which seven were willing to cooperate. We then contacted the participants personally. Of 108 stayers 50 agreed to take part in the study.

Participants were located in all regions of Germany and were interviewed in their homes or local refugee centres. In the majority of the interviews, trained interpreters were employed, allowing the participants to express themselves in their own languages. All participants completed written consent forms, approved but the Konstanz University Ethical Review Board and were assured that all interviews were confidential. The interviews were approximately two hours long. Participants who experienced distress after the interview were referred to local health professionals.

### Participants

For the group of returnees forty-seven refugees from the former Yugoslavia, Iraq and Turkey, who had decided to return with VARP to their home countries, were interviewed by trained interviewers from the Department of Psychology at the University of Konstanz. These three countries were chosen because they had the highest number of voluntary returnees in 2006 [8]. Inclusion criterion was that participants had given a written informed consent form for participation in an assisted program of voluntary return. Also, at their first arrival at Germany they had to have been registered as a refugee. Participants were between the ages of 19 and 90, with an average age of 43. 50.5% were female; the average length of education was 8.5 years. The average duration of stay in Germany was 11.8 years.

The group of stayers consisted of 53 participants, who opted not to return and to remain instead in Germany. Table 1 summarizes further descriptive statistics on these demographic characteristics for each group. Not all participants completed the entire assessment. The reasons for this were emotional distress caused by topics discussed during the interview or severe psychological disability. In four cases only demographic characteristics and information from medical records were included in the database.

**Table 1:** Demographic characteristics

		**Total (n = 100)**	**Returnees (n = 47)**	**Stayers (n = 53)**	**Statistics**	
**Age in y (SD)**	Mean	43.2 (14.9)	48.7 (17.2)	38.3 (10.3)	*t *(98)= -3.6	*p *< .01
**Sex N (%)**	Male	50 (50.0)	25 (53.2)	25 (47.2)		
	Female	50 (50.0)	22 (46.8)	28 (52.8)		
**Country of origin N (%)**	Bosnia	30 (29.7)	9 (19.1)	21 (39.6)		
	Serbia	27 (27.0)	17 (36.2)	10 (18.9)		
	Kosovo	30 (29.7)	11 (23.4)	19 (35.8)		
	Iraq	5 (5.0)	5 (10.6)	-		
	Turkey	8 (8.0)	5 (10.6)	3 (5.7)		
					*Chi square *(4) = 15.0	*p *< .01
**Ethnical group N (%)**	Bosnian	27 (27.0)	8 (17.0)	19 (35.8)		
	Serbian	9 (9.0)	5 (10.6)	4 (7.5)		
	Albanian	19 (19.0)	6 (12.8)	13 (24.5)		
	Roma	25 (25.0)	12 (25.5)	13 (24.5)		
	Kurdish	8 (8.0)	8 (17.0)	-		
	Ashkali	6 (6.0)	6 (12.8)	-		
	Others	6 (6.0)	2 (4.2)	4 (7.5)		
					*Chi square *(8) = 24.8	*p *< .01
**Marital status N (%)**	Single	19 (19.0)	7 (14.9)	12 (22.6)		
	Married	57 (57.0)	29 (61.7)	28 (52.8)		
	Divorced	14 (14.0)	6 (12.8)	8 (15.1)		
	Widowed	10 (10.0)	5 (10.6)	5 (9.4)		
**Duration of stay in Germany in y (SD)**	Minimum	2	3	2		
	Maximum	18	18	16		
	Average	11.8 (4.1)	13.1 (4.2)	10.8 (3.7)	*t*(83)= -2.8	*p *< .01
**Children in school in Germany (%)**	Yes	54 (60.7)	23 (63.9)	31 (58.5)		
	No	45 (39.4)	13 (36.1)	22 (41.5)	*Chi square *(2)= 15.3	*p *< .01
	No answer	11	11	0		
**Education in y (%)**	0–6	29 (32.2)	14 (37.8)	15 (28.3)		
	7–10	26 (28.8)	10 (27.0)	16 (30.8)		
	11–18	35 (38.8)	13 (35.1)	22 (41.6)		
	Missing	10	10	0		
	Average (SD)	8.53 (4.5)	7.7 (4.9)	9.08 (4.2)		

### Data analysis

The data was coded and analyzed using the SPSS package. Descriptive statistics were used to examine the demographic data. Chi-square analyses, Fisher's Exact Tests as well as independent sample T-tests were conducted to determine differences between the study group of 'returnees' and the comparison group of 'stayers'. These statistical analyses were also conducted to determine differences within the group of 'returnees', i.e. between men and women or between participants with or without mental disorders. Correlations between different aspects such as integration, willingness to return, mental health, education and quality of life were tested by calculating coefficients such as Spearman and Pearson depending on the particular data.

Due to the fact that some participants refused -or were not able- to answer certain questions, the sample size varies between interview sections. For this reason the sample size is reported in each analysis.

### Outcome measures

#### Demographics and return

This questionnaire was designed to collect information on the living conditions of the participants. It includes questions regarding origin, ethnicity and religion, age, sex, marital status, level of education, employment and clinical history. Further questions are concerned with the reasons for and circumstances of the participant's flight, duration of stay in Germany and current living situation in Germany. The degree of integration was determined based on theoretical considerations by Heckmann [23]. Heckmann says there are three levels of integration which must be considered.

The 'structural' level includes aspects such as the status of one's residence permit, work permit and employment situation. The 'social level' describes how the subject divides their leisure time and how much of that time is spent with Germans, with people from their own country of origin or alone.

Finally, the 'cultural level' looks at knowledge of German language and how connected the subject feels to Germany and to their country of origin respectively. Subjects were questioned about each of these levels. Their answers were rated on a 3-point Likert scale ranging from 0 (no integration) to 2 (good integration). The sum derived from all categories constitutes the integration index.

Another section of the questionnaire analyses what motivates participants to opt for or against voluntary return to their country of origin. According to Gmelch, these motivators were divided into three categories: 'familial-personal reasons', 'economic-occupational reasons' and 'social-patriotic reasons' [21]. Along with all reasons reported by the participants, what they identified as the most important motive was registered separately in an attempt to assess the key motive that has lead to the decision in each individual case [22].

#### Posttraumatic stress

The Posttraumatic Stress Diagnostic Scale (PDS) was used to assess symptoms of posttraumatic stress. The scale is a self report questionnaire which is designed to aid in the detection and diagnosis of PTSD [[19], German version: 15]. It consists of a traumatic event scale and a symptom scale. The symptom scale consists of 17 items which comprise the subscales 'intrusions', 'avoidance', and 'hyperarousal'. It is closely oriented on the DSM-IV criteria for PTSD and may be administered repeatedly over time to help monitor changes in symptoms. Participants were asked to indicate the frequency of each symptom over the four weeks prior to the interview on a 4-point Likert scale, with 0 meaning 'Not at all or only one time' and 3 meaning 'Five or more times per week/almost always'. In the present study we used the PDS as an interview.

#### Mental health

Psychological functioning was measured using the German version of the Mini International Neuropsychiatric Interview (M.I.N.I.), Version 5.0.0 [[43], German version: 1]. The M.I.N.I. is a short structured diagnostic interview for DSM-IV and ICD-10 psychiatric disorders. Participants were asked to indicate which of the symptoms they had experienced within the four weeks prior to the interview. Validation studies [43] have shown good validity and reliability in making diagnoses in less time than conventional structured interviews such as the SCID-P or the CIDI. In this study the sections I (PTSD), L (psychotic disorders) and P (antisocial personality disorder) were not included in the interview.

#### Quality of life

The EUROHIS-QOL 8-item index is a subjective measure for Quality of life (QoL), derived from the WHOQOL-100 and the WHOQOL-BREF [59]. The overall QoL score is formed by a summation of scores from the 8 items, with higher scores indicating better QoL. Conceptually the four domains measured (psychological, physical, social and environmental) are each represented by two items. Each item can be answered on a 5-point Likert scale ranging for instance from 1 (not at all) to 5 (completely). A study by Schmidt et al. has revealed a good reliability and validity of the measure across a range of countries [42].

## Results

### Mental health

In 21 returnees (43.8%) we found at least one or more mental disorders according to DSM-IV criteria (*n *= 47). As shown in Table 2, PTSD was detected most frequently, followed by affective disorders and anxiety disorders. Suicidal tendencies were also detected in high rates. In comparison to the group of stayers, where the prevalence of mental disorders was 78%, it was lower among returnees. However, their rates remain considerably higher than in the average population in Western countries (Kessler, 1995; Maercker, 2007). 38.3% of the returnees consulted a psychotherapist and/or psychiatrist, compared with 58% of stayers.

**Table 2:** Mental health in refugees in Germany in %

**Mental disorder**	**Returnees (N = 47)**	**Stayers (N = 53)**
**At least one DSM-IV diagnosis**	43.8	78.0
**PTSD**	31.0 (n = 42)	54.7
**Depression**	31.9	51.9
**Manic Episode**	-	1.9
**Dysthymia**	10.6	13.5
**Suicidal Tendencies**	27.7 (low)	43.2 (low to high)
**Psychotic Disorder**	8.5	(not explored)
**Agoraphobia**	8.5	9.6
**Panic Disorder**	6.4	9.6
**Social Phobia**	-	9.6
**Obsessive-Compulsive Disorder**	-	-
**Eating Disorder**	-	3.9
**Substance abuse/dependence**	2.1	1.9
**General Anxiety Disorder**	4.3	-

**Undergoing psychological treatment**	38.3	58.5

31% of the returnees developed PTSD. This figure does not include the large number of returnees who had experienced traumatic events which did not develop into PTSD. Among the 94 traumatic events reported by returnees (*n *= 43), experiences related to war and violence occurred most frequently (30.4% war-related events, 28.3% related to the witnessing of a violent attack, 26.1% experiences of violence against one's own person).

Statistical analysis showed that the degree of voluntariness had no impact on mental health. Also in our study we did not find any particular factors, such as gender, age or marital status, which in other studies have been proven to be risk factors for mental disorders.

### Subjective quality of life

Subjective quality of life (QoL) was measured on a scale from 1 to 5. The average value among returnees was *m *= 3.13 (*SD *= .78, *n *= 41). Statistical analysis showed no significant difference between mentally healthy participants and participants with at least one mental disorder. In contrast, in the group of stayers, QoL differed significantly between healthy subjects (*m *= 4.03, *SD *= .59, *n *= 53) and those who had at least one mental disorder (*m *= 2.77, *SD *= .68, *t *(37.4) = 5.65, *p *< .01).

A significant negative correlation was found between age and QoL (*r *= .-39, *p *< .05).

### Integration

Integration was measured on a scale from 0 to 12. The integration index in the group of returnees was *m *= 4.74 (*SD *= 2.1, *n *= 43). The index of integration did not change significantly when excluding participants from Turkey and Iraq. The level of integration among stayers was significantly higher (*t *(94) = 2.4, *p *< .05) with an average of *m *= 5.9 (*SD *= 2.6, *n *= 53). As shown in Table 1, the average duration of stay in Germany at the time of the interview was 13.1 years (*SD *= 4.2) among returnees and 10.8 years (*SD *= 3.7) among stayers.

The significantly lower level of integration among returnees can be explained by more unfavourable living conditions. As the analysis revealed, this includes all aspects of integration evaluated in this study (in brackets the corresponding figures for the group of stayers are presented):

#### Cultural level

31.9% (56) spoke fluent German. 47.8% (82) felt more at home in Germany than in their country of origin.

#### Social level

84.8% (64) limited their social activities to family and household, while 13% (40) had more contact with Germans in Germany than with people from their country of origin.

#### Structural level

97.8% (90.1) had a limited residence permit. Of there, 23.4% (7.5) had to leave the country within 4 weeks, 17% (22) had a legal tenure, while 17% (18) had a work permit, but no tenure. Another 4.3% (24) were working without official permission.

Statistical analysis showed that being employed, whether legally or illegally, resulted in better integration (*t *(24) = -6.1, *p *< .01).

Looking at integration from the perspective of education reveals another important finding: Participants with a higher level of education (> 8 years in school) were significantly better integrated (*t *(36) = 4.1, *p *< .01). Data also shows that the rate of employment among participants with more education was significantly higher (*t *(34) = 4.7, *p *< .01). Women in the study tended to be significantly less educated than men (*t *(31) = 2.4, *p *< .05) and elderly people (> 60) less educated than young participants (*r *= -.46, *p *< .01). Also there was a negative correlation between integration and age (*r *= -.37, *p *< .02), which could not be found for integration and female sex, even though the integration index of women was lower (*m *= 4.1, *SD *= 1.6) than of men (*m *= 4.5, *SD *= 1.9).

### Return

All participants who were referred to us had signed a written consent form at the immigration office in which they agreed to return voluntarily to their home country. In order to allow for a better understanding of our findings we have divided the following section into three parts: a) *motives for and against voluntary return*, b) *voluntariness of the decision to return *and c) *willingness to return under the current circumstances*.

*a) *According to studies by Toren and Gmelch participants spontaneously mention a great number of *motives for and against voluntary return *[21,51]. Table 3 charts how frequently each motive for or against voluntary return was reported in the group of returnees (*n *= 43). The contra-motive "security and safety" was mainly reported by participants from Iraq (five from Iraq and two from Kosovo). For all other motives the country of origin did not play a role.

**Table 3:** Reasons for and against voluntary return (multiple answers were permitted)

**Motives against a return**	**N**	**%**	**Motives for a return**	**N**	**%**
**Economical reasons**					
Transport/travel cost	2	4.9	Unemployment in Germany	8	19.5
Unemployment in country of return	9	22.0	Dependence on welfare system in Germany	3	7.3
Lack of seed capital	12	29.3			
**Political/social reasons**					
Security and safety	7	17.1	Lack of cultural attachment in Germany	3	7.3
Fear of rejection by people, who stayed in the country	1	2.4	Racism in Germany	1	2.4
Hatred between ethnic groups/discrimination	1	2.4	Poor living conditions in Germany	1	2.4
Housing conditions	7	17.1			
Poor living conditions in country of return	9	22.0			
Lack of medical and psychological care in country of origin	13	31.7			
**Personal/familial reasons**					
Personal attachment to Germany	5	12.2	Family ties in home country	18	43.9
Children born and raised in Germany	14	34.1	Homesickness	22	53.7
Personal failure	1	2.4	Cultural roots	7	17.1
Fear of confrontation with traumatic context	1	2.4	Independence in home country/language skills	2	4.9
Better prospects for children in Germany	4	9.8	Wish to die in home country	8	19.5
			Fear of forced deportation	10	24.5

When asked for their personal key motive in the consideration of a voluntary return, the contra-motive 'children born and raised in Germany' was reported most often (14.9%), followed by the contra-motive 'lack of medical and psychological care in country of origin' (8.5%) and the pro-motive 'Wish to die in home country' (8.5%).

*b) *The discrepant finding that such a great number of participants mentioned contra-arguments while having signed an informed consent for a voluntary return required further analysis. Thus, participants were asked about the influences that have led to the *decision to return*. 55.3% reported that they had experienced pressure from state officials and/or the immigration department which included the alternative to be returned under duress. Another 6.4% reported that they were pressurized by their partner, who had already been deported to the country of origin. That is, in 61.7% of the returnees the decision to return was not voluntary or was strongly influenced by external factors.

*c) *Due to the strong influence of external factors on the decision to return and the clear divergence between subjective opinion and objective circumstances in the environment of the returnees, they were then asked to describe and rate their willingness to return considering their current situation. Results were measured on a 10- point Likert scale (1 = I absolutely do not want to return, 10 = I absolutely want to return). Willingness to return voluntarily was very low in people who return under the influence of external pressure. It was high in people whose decision to return was an expression of their own will. Explanations for very low values (1–3) were fear of an uncertain future in the country of origin as well as fear to be forced to leave Germany under duress. Participants who chose high values (8–10) explained that their decision was influenced by the wish to die in their home country or to live with their spouses, who had already been expelled from Germany. It is important to note that some participants reported a maximum *willingness *to return while at the same time stating that the *decision *to return was involuntary. Explanations given here were: "Since I have to leave, I will accept the decision and try to make the best of it." or: "I choose 10 under the condition that I really get medical, financial and social support as promised in the return program".

Statistical analysis allowed for further understanding of these statements. Participants with a high or low willingness to return, respectively, differ in two aspects: First, willingness to return rises with the degree of homesickness (*r *= .62, *p *< .01) and secondly, participants who had visited their home country after their departure expressed a significantly higher willingness to return (*t *(37) = -5.9, *p *< .01).

Eleven participants dropped out of the program before return (with four of them having reported a high willingness to return). Reasons were the achievement of permanent residential status in Germany, an unwillingness to cooperate in the preparation of the return and/or dissatisfaction with the financial support offered by the program.

There were eleven participants who reported a high willingness to return, reported that their decision was voluntary and actually returned to their home country. Of these eleven, eight participants (73%) were older than 70 or were ill with cancer and wanted to die in their home country. These results are congruent with the statistical finding that willingness to return was significantly higher with age (*r *= .49, *p *< .05).

## Discussion and Conclusion

### Mental health

The results of the study presented in this paper show that psychological distress among participants of VARP, who once arrived at Germany as refugees, is high before the return. Even though prevalence of mental disorders is lower than in a comparable sample of refugees who decided to stay in exile (stayers), it is significantly higher than in the average population of Western countries.

As demonstrated in earlier studies on refugee populations, the most frequent diagnosis is PTSD, followed by affective disorders and anxiety disorders. Rates of suicidal tendency in the described population are elevated in comparison to the average population as well.

There are multiple causes for the development of mental disorders, making it difficult to trace their roots. One exception is PTSD, where aetiology is part of the diagnostic criteria. Earlier studies with a prospective design have demonstrated that traumatic events can also result in mental disorders other than PTSD [61]. Considering that all but four of the respondents reported experiences of traumatic events in the past, traumatic stress can be accounted for as one cause for mental disorders other than PTSD in our group, too. On the other hand, there are other stressors in exile, which are permanently present such as language barriers, unstable residence status, being blocked from working etc. Such factors are known to promote mental ill-health and are therefore likely to have contributed to the high prevalence of psychological disorders in both groups.

### Integration

The index for integration of refugees in Germany, who are going to return to their country of origin, was low. Considering that the average duration of stay was 13 years, this low index is remarkable. An important reason for this result is the high percentage of participants with unstable residential status which prohibits people from working legally. Also, a quarter of the group was under the pressure to leave the country within the next month. Interestingly the group of stayers was significantly better integrated even though the percentage of unstable residence situations was almost as high as in the group of returnees. Thus, other factors must be responsible for the difference between groups. These are, according to our analyses, work status, age and education. In the group of stayers a higher number of people were working, whether their permit allowed for this or not. This probably opened up more possibilities to integrate into the host society. On the other hand, returnees were significantly older on average, which promotes a number of difficulties. Thus, with advanced age it is more difficult to integrate just as it is more difficult to find work, to learn a new language, to contact other people or to simply move around in the environment without physical limitations. They also tend not to have children in school – a place which would provide another point of contact with the host society.

Regardless of age, education played an important role for integration in the group of returnees. Again, older people were disadvantaged here, as many of them grew up in rural areas where access to education was either limited or where the school system was not yet as developed as it may be today. In addition, in terms of education, women are more disadvantaged than men. This finding is notable if education turns out to be a key factor for integration. Overall the findings on integration provide us with two conclusions. First, a large number of refugees in Germany are isolated from German society even though they have been living here for an extended period of time. This affects the mental health and wellbeing of the people concerned. Besides the problematic humanitarian consequences of a situation in which migrants are isolated from the host society it also means a loss for the receiving country. Migrants are entering the country with a number of skills and often a high motivation to work from which the receiving country could benefit if these people would be allowed to work and to integrate in society. Second, the intention to prevent migrants from integration and to promote voluntary return by means of political instruments (such as prolonged unstable visa status, the prohibition to work or financial incentives for returnees) is not successful. The vast majority of refugees prefer to stay in Germany no matter under which conditions. That means that neither 'Push Factors' in the form of poor living conditions and low level of integration nor money offered by voluntary return programs seem to be factors that are strong enough to persuade people to leave safe countries such as Germany.

### Subjective quality of life

The results show that returnees in this sample rated their quality of life as moderate. Compared to the index of healthy people in 10 western countries (which amounts to *m *= 3.68, *SD *= 0.62) no large differences were found [42]. It is possible that when answering questions on quality of life, refugees (returnees as well as stayers) compared their living situation to the one in their home country, which they found comparatively worse. When considering the degree of danger during times of war, as well as the living standard of people who remained in the home country during and after wartime, this perception seems very realistic. Among returnees, the quality of life of people older than 60 was lower than in young participants. Because of their age, they are confronted with additional difficulties such as reduced mobility, social isolation and poor health – factors which also contribute to the low level of integration among elderly participants.

Healthy returnees and those with at least one mental disorder did not differ greatly from each other in terms of their subjective quality of life. In stayers, however, there was a statistical difference between healthy and ill participants. Probably returnees are in a heightened state of stress and therefore their health may be of less importance to them compared to the voluntary or forced return to their home country which is in each case a major distressing life event.

### Return

The findings regarding participants' attitude towards 'voluntary return' turned out to be complex. There is a large discrepancy between participant opinions towards voluntary return and the facts they are confronted with by German immigration authorities. In the study we therefore had to differentiate between general attitudes for and against voluntary return, voluntariness of the decision to return and willingness to return under the current circumstances.

When asked about their general attitude towards voluntary return, homesickness was mentioned most frequently as an aspect which made people consider returning. But even though participants seemed to miss their home country very much, this was not the activating key motive which made them opt to return.

More than any other reason, participants cited their children's futures as the key motive in the decision. However, this motive was actually against return, which at first sight is surprising in a group of voluntary returnees. It is less surprising once the *voluntariness of the decision to return *is included in the analysis. The majority of the participants made their decision under external pressure such as the threat of deportation. This fact contradicts the requirement of voluntary assisted return programs, in which the decision is supposed to be voluntary. It also explains why there is a subgroup of refugees from Iraq in our study who are participating in voluntary return programmes even though they are expressing a fear to return because of the ongoing war in their country of origin. At this point it is important to consider which definition of voluntariness is applied.

As cited in the introduction, in the UNHCR definition external pressure and the lack of permanent residential status – as found in the majority of our sample – are contradictory to a voluntary decision as well as a voluntary return. In contrast, in the definition of IOM the requirement of voluntariness is already fulfilled with the absence of active resistance during the actual return process. The decision process is not included here at all. German immigration authorities and organisations concerned with voluntary assisted return, define their activities based on the definition of IOM. Clinical psychologists, whose interventions are based on the experience and behaviour of the individual, can relate more easily to the definition by the UNCHR as it puts more emphasis on the subjective perspective of the people concerned. From that perspective for the majority of our participants the requirement of voluntariness is not fulfilled.

In our opinion in the discussion about 'voluntary assisted return' it is very important to differentiate between the different players involved. National Voluntary Return Programs are a political instrument which is implemented by receiving countries. On the one hand they are designed for the benefit of the implementing country – they reduce the number of dependents on the welfare system and send a clear signal to future migrants. They can also provide a humanitarian solution to the situation of refugees who will have to return to their country of origin in the near future. But in comparison to this political perspective the actual decision to return affects the individual on a personal level including the life changing consequences following it. So, it seems very difficult to meet the interests of both -those who imply the programs and those who the programs are made for- at the same time. This may be one reason why the assessment of VARPs differs so strongly depending on the perspective of the evaluator.

An often overlooked fact in the debate at hand is the duration of stay in the Receiving Country. Refugees in Germany are usually in flight for political reasons – which is why Germany accepted them. Whereas immigration policy only considers one's motives at the time of arrival in Germany, in many people reasons for a life away from the country of origin change overtime.

After the flight refugees are trying to continue their life in exile, to tie up to former plans or to realize new live models. Children are born, families grow and people adjust to their new environment. Upon closer examination, we see that it is even more complex. The participants of our study lived in Germany for more than a decade, with only temporary residential status and all the insecurities that come with such status. They spent this time waiting for a decision about where their lives would continue, all the while adapting to Germany and becoming more distanced from their country of origin. In this sense, the aftermath of war continues. Moreover, their home country has often undergone massive changes. For example, the former Yugoslavia, a socialist republic, has divided into several nation states with a free market economy. They lost their possessions and often family members. Often, those who did not leave during the war are blaming those who are returning now for having escaped and benefited from richer countries. At the same time, in Germany, they are only accepted temporarily and prevented from integrating and starting a new live for themselves. These people find themselves in a no-win situation, and many reported they no longer expected much from their own lives. In this situation they make the decision based on the needs of their children. So, a key motive in the decision to return concerns children born and raised in Germany. Despite strong pull factors like 'family ties in the home country' and 'homesickness' as well as strong push factors like 'temporary residential status' and 'prohibition to work', people decide to stay for their children. Supposedly, other factors, such as the higher standard of living in Germany also play an important role. But interestingly, neither these nor worries regarding the living situation in the home country were mentioned. Even the fear of a confrontation with traumatic stressors in the homeland does not seem to play a role at all in the group. Considering the high prevalence of PTSD in the group this result is significant. It seems that after 13 years in exile refugees do not take on the perspective of the home country even when it comes to return.

To sum up, the abolition of the Push Factor war does not automatically incite a desire to return in all refugees. Considering that participants spent some 13 years of their life time in Germany it can be better understood that reasons against a return change from the political to the personal level. Across such long time periods, we would expect that differences between stayers and returnees will level out, which would explain the similarity between both groups in the present investigation.

An important observation for policy makers may be that people who reported a high *willingness to return *were those who had visited their homeland after the conflict and crisis and those who were significantly more homesick. Restrictions in travel permission to refugees with temporary residence status as it seems are neither humanitarian nor do they increase the willingness to return.

Finally, we want to pose the question: who are the people willing to return voluntarily? Eleven participants expressed voluntariness and a high willingness to return. Their reasons were homesickness, the expectation of support from the return program and acceptance that one has to leave and to make the best of it. In the end, four of them did not leave Germany. According to our data, those who really returned voluntarily were older than 70 or terminally ill and had a desire to die and be buried in their homeland. As demonstrated in several sections of this study, old people compose a special subgroup among returnees. On the one hand, their level of integration as well as their subjective quality of life tends to be lower. At the same time, they fulfil the criteria of a voluntary return. Left aside that more efforts should be made to integrate elderly migrants into German society for humanitarian reasons alone, Assisted Voluntary Return Programs could create specific guidelines for handling this group. In contrast to all other returnees these elderly people were the only participants in our study who left Germany as truly voluntary returnees.

Among the limitations of our study is the size of the studied sample. Participants were recruited through all varieties of programs dealing with assisted 'voluntary' return in Germany. Despite extensive efforts, the group of returnees examined for this report is relatively small. In cases of low feedback, organisations which did not refer clients to us were asked to explain why. Four organisations did not want to cooperate for fear the data could be misused for political reasons such as a campaign for or against the practice of VARP. Other organisations argued that the interview would be stressful for their clients (especially when a mental disorder has already been diagnosed). In some refugees the fear of the return was very high and organisations were not willing to refer them to the study. Interestingly, the majority of organisations did not have any clients who fit our inclusion criteria. They reported to have very few clients in general as the demand for VARP was very low during the study period.

Unfortunately there is very little information on the mental health of refugees in Germany which makes it difficult to estimate the representativeness of our results. This in fact stimulated us to perform this study. The only data which exists in Germany comes from an earlier study, which found a PTSD-prevalence rate of 40% among asylum seekers in Germany [20]. This is comparable to the PTSD-rates found in the present study. As far as we know there is no data available on demographic characteristics of refugees living in Germany. For returnees we also could not obtain general, i.e. nation-wide, information on demographic characteristics. Therefore we examined the demographic statistics of those organisations involved in VARP [10,11,24]. According to these statistics our sample is representative for returnees in Germany regarding age, gender, marital status, residence status and country of origin.

Given that the study includes returnees from a variety of organisations, particularly those, which are more confident in their programs, effects for all the organisations might be even stronger and the current conclusions are likely to be valid. The number of people with a high degree of mental illness is not rare among returnees. For reasons of representativeness we included such participants as well, even though they were not all able to complete the whole interview. The group of returnees from Turkey and Iraq in the present sample is small. For this reason, these groups were not examined independently from the large group of participants from former Yugoslavia. Statistical analysis excluding participants from these two countries came to the same results as when they were included in the analysis.

Taking into consideration the general lack of information on refugees in Germany we limit our findings to the group of returnees in Germany who came as refugees and are returning now with assisted programs of voluntary return. Within that frame our findings are representative. In terms of voluntary return in Germany in general our study has the character of a pilot study. Further investigations with larger samples from different countries of origin should be performed to assure and deepen the results of the study presented in this paper.

The debate on mental health among refugees often addresses how much importance should be given to simulation, i.e. the assumption that refugees with uncertain residence status are simulating a negative mental state in order to avoid a forced return. If simulation or aggravations play an important role prevalence rates reported in this study would have to be questioned. Ruf et al. conducted a longitudinal study examining refugees diagnosed with PTSD and depression before and after they had received a permanent visa. The study reveals a decrease in depression but not of PTSD in the second assessment [41]. Also participants in our study had already commenced the return process and would not benefit from aggravation. We therefore assume that the described prevalence rates are realistic.

Since a number of organisations did not refer clients to us who have already been diagnosed with a mental disorder, we suppose that the prevalence of mental disorders among returnees is probably higher than presented in this study. In this respect, the numbers presented here can probably be interpreted as a conservative estimate.

Another limitation of the study is the lack of validated translations of the applied questionnaires. In order to compensate for this problem we used instruments which have been translated back and forth by clinical psychologists in Germany and the countries of origin. Also we employed interpreters who were experienced translating in a clinical setting as well as in the use of structured interviews and gave them further training for the instruments used in our study. For further investigation it would be recommendable to apply questionnaires which are validated in the respective language.

Regarding motivations for and against return, this study shows that refugees consider return from many perspectives. Even though prevalence rates of mental disorders are highly elevated in returnees in comparison to the average population, their mental health status does not seem to play a key role in the decision. Considering that for the majority the decision to return is strongly influenced by external factors such as the unstable residential status it is clear why the political level is so much more influential in the return process than the personal level.

Nonetheless, it is important to study the phenomenon of return from a psychological and individual perspective: Mental health is not only relevant from a humanitarian perspective, where it is a prerequisite for individual wellbeing. It is also a key in achieving successful reintegration. Clinical studies show that treatment opportunities in the countries included in our study are limited and expensive. Sometimes, they do not exist at all. The cost of medication is often prohibitive. Due to symptoms related to their diagnosis (such as withdrawal, sadness, distrust etc.) people with mental disorders encounter far more integration challenges. This is especially difficult in people with PTSD as their symptoms include the fear of all triggers related to the original trauma and a lack of a perspective for the future. These considerations touch the political-societal perspective as well, as a post war society as a whole benefits from the successful reintegration of former refugees. It is obviously important to have healthy citizens in a post-war society in order to rebuild the nation physically and politically. However, the limited number of studies in this field makes it difficult to frame a prognosis. Certain stressors which have been influential in exile become less important – such as homesickness or inability to attain a work permit-, while new stressors could turn up after the return – economical insecurity, being stranger in their own country, confrontation with triggers of traumatic experiences. To get a better picture of mental health of refugees in the return process, the participants of this study will be interviewed again nine months after return.

## Abbreviations

BAMF: Bundesamt für Migration und Flüchtlinge (Federal Office for Migration and Refugees); EUROHIS: Health Interview Survey in Europe; IOM: International Organisation for Migration; M.I.N.I.: Mini International Neuropsychiatric Interview; PDS: Posttraumtic Stress Diagnostic Scale; PTSD: Posttraumatic Stress Disorder; QoL: Quality of Life; SPSS: Statistical Package for the Social Sciences; UNHCR: United Nations High Commissioner for Refugees; VARP: Voluntary Assisted Return Program; WHO: World Health Organisation; ZIRF: Zentralstelle für Informationsvermittlung zur Rückkehrförderung (Central Information Office on Assisted Return).

## Competing interests

The authors declare that they have no competing interests.

## Authors' contributions

Concept: UvL, FN, TE; Data searching: UvL, UW; Analysis: UvL, UW; First draft: UvL, UW; Critical revisions: UvL, UW, TE, FN; Final manuscript read and approved: UvL, UW, TE, FN.

## Pre-publication history

The pre-publication history for this paper can be accessed here:


